# Draft Genome Sequence of Sulfurospirillum sp. Strain MES, Reconstructed from the Metagenome of a Microbial Electrosynthesis System

**DOI:** 10.1128/genomeA.01336-14

**Published:** 2015-01-15

**Authors:** Daniel E. Ross, Christopher W. Marshall, Harold D. May, R. Sean Norman

**Affiliations:** a Department of Environmental Health Sciences, University of South Carolina, Columbia, South Carolina, USA; b Department of Microbiology & Immunology, Marine Biomedicine & Environmental Science Center, Medical University of South Carolina, Charleston, South Carolina, USA

## Abstract

A draft genome of Sulfurospirillum sp. strain MES was isolated through taxonomic binning of a metagenome sequenced from a microbial electrosynthesis system (MES) actively producing acetate and hydrogen. The genome contains the *nosZDFLY* genes, which are involved in nitrous oxide reduction, suggesting the potential role of this strain in denitrification.

## GENOME ANNOUNCEMENT

Microbial electrosynthesis is a recently discovered process whereby microorganisms consume electricity and CO_2_ for the production of reduced end products, such as commodity chemicals and fuels. Electrochemical data have suggested direct and/or mediated electron transfer between the electrode and biocatalyst, but the specific metabolic pathways involved remain poorly understood. The draft genome described here was obtained from shotgun sequencing of an electroacetogenic mixed-microbial community metagenome. The microbial electrosynthesis system (MES) was operated for >150 days with a cathode potential of −590 mV versus standard hydrogen electrode (SHE) (1, 2).

A dual sequencing approach was utilized for the aforementioned MES metagenome. Approximately 32 million and 900,000 reads were generated using the Illumina MiSeq platform (2 × 250-bp paired-end sequencing) and the Pacific Biosciences (PacBio RS) platform, respectively. The Illumina reads were quality trimmed with the CLC Genomics Workbench. The trimmed Illumina reads were utilized for subsequent genome assembly and error correction of the PacBio reads. The error-corrected PacBio reads and trimmed Illumina reads were assembled with Velvet, and the contigs were binned by phylogeny using BLASTn (3). The Sulfurospirillum-associated contig bin was used to map the raw metagenome reads. The resultant Sulfurospirillum-associated Illumina and PacBio reads were assembled using a combination of Velvet, CLC Genomics, and SPAdes (4, 5). The draft genome of this strain, thus named Sulfurospirillum sp. strain MES, is 2.67 Mbp (G+C content, 43.8%) contained in 130 contigs (61 >500 bp), with an *N*_50_ of 371,847 bp and the longest contig being 724,139 bp. The scaffolds were annotated with RAST (6), revealing a total of 2,691 features (2,655 protein-coding genes and 36 RNAs).

Phylogenetic analysis of the full-length 16S rRNA gene from the Sulfurospirillum-associated genome bin suggests that the Sulfurospirillum sp. strain MES is most closely related to the cultured Sulfurospirillum cavolei strain Phe91 (99% identical) and uncultured/enrichment culture clones associated with wastewater-activated sludge and petroleum reservoirs (7). An initial genome-wide comparison to other Sulfurospirillum organisms with a draft or complete genome sequence suggests that Sulfurospirillum sp. strain MES is most closely related to Sulfurospirillum multivorans (81.6% and 78.0% identical at the nucleotide and amino acid levels, respectively). The members of Sulfurospirillum are heterotrophic denitrifiers capable of dissimilatory selenite and arsenate reduction and have been found in oil fields, aquifer sediments, and contaminated groundwater (8–10). Sulfurospirillum organisms have metabolic capabilities associated with reductive dehalogenation (10–12). These metabolisms are retained in the draft genome, which codes for the ability to utilize a wide variety of electron acceptors (e.g., arsenate, sulfur, and nitrate) and electron donors (e.g., formate and hydrogen [in the presence of acetate], pyruvate, fumarate, and lactate). Pangenomic analysis and comparative genomics are under way to analyze the other sequenced Sulfurospirillum genomes and to determine the importance of Sulfurospirillum sp. strain MES within the acetogenic MES. The aforementioned genome data provide new genetic insight into the Sulfurospirillum genus.

### Nucleotide sequence accession numbers.

The Sulfurospirillum sp. strain MES draft genome has been deposited at GenBank under the accession no. JSEC00000000. The version described in this paper is version JSEC00000000.
